# Estradiol regulates voltage-gated potassium currents in corticotropin-releasing hormone neurons

**DOI:** 10.1242/jeb.245222

**Published:** 2023-03-10

**Authors:** Emmet M. Power, Dharshini Ganeshan, Karl J. Iremonger

**Affiliations:** Centre for Neuroendocrinology, Department of Physiology, School of Biomedical Sciences, University of Otago, Dunedin 9016, New Zealand

**Keywords:** CRH, Estradiol, Ion channel, Intrinsic excitability, Ovariectomy, M-type potassium channel, A-type potassium channel

## Abstract

Corticotropin-releasing hormone (CRH) neurons are the primary neural population controlling the hypothalamic–pituitary–adrenal (HPA) axis and the secretion of adrenal stress hormones. Previous work has demonstrated that stress hormone secretion can be regulated by circulating levels of estradiol. However, the effect of estradiol on CRH neuron excitability is less clear. Here, we show that chronic estradiol replacement following ovariectomy increases two types of potassium channel currents in CRH neurons: fast inactivating voltage-gated A-type K^+^ channel currents (*I*_A_) and non-inactivating M-type K^+^ channel currents (*I*_M_). Despite the increase in K^+^ currents following estradiol replacement, there was no overall change in CRH neuron spiking excitability assessed with either frequency–current curves or current ramps. Together, these data reveal a complex picture whereby ovariectomy and estradiol replacement differentially modulate distinct aspects of CRH neuron and HPA axis function.

## Teaser

**Summary:** Chronic estradiol replacement in ovariectomized mice influences voltage-gated potassium channel function.

## INTRODUCTION

Corticotropin-releasing hormone (CRH) neurons in the paraventricular nucleus (PVN) of the hypothalamus are neuroendocrine neurons that control activity of the hypothalamic–pituitary–adrenal (HPA) axis (Herman and Cullinan, 1997; Ulrich-Lai and Herman, 2009; Kim et al., 2019a,b; Füzesi et al., 2016; Sterley et al., 2018; Daviu et al., 2020). These neurons are activated in response to stress (Kim et al., 2019b), which leads to CRH secretion from the median eminence into the portal circulation. This triggers secretion of adrenocorticotropic hormone (ACTH) from the anterior pituitary, which subsequently stimulates corticosteroid synthesis and release from the adrenal cortex.

Activity of the HPA axis is sexually dimorphic. In rodents, females have higher levels of circulating corticosterone as well as stress-evoked corticosterone release (Seale et al., 2004). There are also marked changes in activity of the HPA axis across the female reproductive cycle, with both basal and stress-evoked levels of corticosterone being highest on proestrus (Atkinson and Waddell, 1997; Viau and Meaney, 1991). Because estradiol is a primary sex hormone in females and is at its highest levels on proestrus, this has led to the theory that estradiol could be responsible for both sex and estrous cycle differences in HPA axis activity (Nilsson et al., 2015). Consistent with this idea, basal corticosterone secretion in female rats is reduced following ovariectomy (Babb et al., 2013; Seale et al., 2004; Young et al., 2001). Circulating corticosterone levels can also be elevated in ovariectomized (Ovx) rats with subsequent estradiol replacement (Figueiredo et al., 2007; Kitay, 1966; Lo et al., 2000). Despite this, other data in rats show that estradiol suppresses stress-evoked ACTH release (Babb et al., 2013; Young et al., 2001) as well as stress-evoked cFos labelling in CRH neurons (Dayas et al., 2000; Figueiredo et al., 2007; Gerrits et al., 2005). To add to this complex picture, studies investigating the effect of estradiol replacement in Ovx mice are conflicting. Some studies report that estradiol replacement in Ovx mice can increase corticosterone levels (Kreisman et al., 2020), whereas others report no effect (Aoki et al., 2010; Speert et al., 2002; Wada et al., 2018) or even reduced corticosterone (Daodee et al., 2022; Eid et al., 2020; Tantipongpiradet et al., 2019; Ghobadi et al., 2016; Tang et al., 2005). Overall, the impact of estradiol on HPA axis function and CRH neuron activity is complex and may differ between species.

We have recently shown that K^+^ channel function and CRH neuron excitability are regulated over the estrous cycle in mice (Power and Iremonger, 2021). During the proestrus phase of the estrous cycle, coinciding with a peak in estradiol levels, CRH neurons exhibit smaller K^+^ channel currents and higher levels of excitability measured by electrophysiological recordings. This corresponds with previous publications showing basal and stress-evoked corticosterone levels being highest during proestrus (Atkinson and Waddell, 1997; Viau and Meaney, 1991). However, it is currently unclear whether these changes in excitability are driven by estradiol alone. Previous work has shown that estradiol can regulate K^+^ currents and excitability in other central neurons (Arroyo et al., 2011; Vastagh et al., 2019; DeFazio and Moenter, 2002). Therefore, in the present study, we aimed to determine the effect of Ovx and high or low levels of estradiol replacement on K^+^ channel function and CRH neural excitability. Using patch-clamp recordings from CRH neurons, we show that chronic elevations in estradiol levels in Ovx female mice leads to increased levels of two K^+^ channel currents: fast inactivating voltage-gated A-type K^+^ channel currents (*I*_A_) and non-inactivating M-type K^+^ channel currents (*I*_M_). However, chronic estradiol elevations did not significantly change intrinsic excitability compared with Ovx animals. This suggests that the magnitude of changes in K^+^ channel currents were not sufficient to impact spiking excitability. Despite this, we speculate that enhanced K^+^ channel function may affect how these neurons integrate and process stress-relevant synaptic inputs.

## MATERIALS AND METHODS

### Animals

All electrophysiological experiments were carried out in adult female (2–6 months old) Crh-IRES-Cre;Ai14 (tdTomato) mice. These mice were generated by crossing the Crh-IRES-Cre (B6(CG)-*Crh^tm1(cre)Zjh^*/J) (Taniguchi et al., 2011) strain with the Ai14 (B6.Cg-*Gt(ROSA)26Sor^tm14(CAG-tdTomto)Hze^*/J) strain, both originally obtained from The Jackson Laboratory (stock numbers 012704 and 007914, respectively). These mice have been previously shown to faithfully label CRH neurons in the PVN (Chen et al., 2015; Wamsteeker-Cusulin et al., 2013; Jamieson et al., 2017). Serum corticosterone and tissue samples were taken from a mixture of C57/Bl6J (The Jackson Laboratory) and Crh-IRES-Cre mice (2–4 months). Animals were subjected to a 12 h:12 h light:dark cycle (07:00–19:00 h lights on) with food and water available *ad libitum*. All protocols and procedures were approved by the University of Otago Animal Ethics Committee and carried out in accordance with the New Zealand Animal Welfare Act.

### Ovariectomy and hormone replacement

Adult female mice (>3 months) were bilaterally ovariectomized under isoflurane general anesthetic. Simultaneously mice received a 10 mm long silastic capsule (inner diameter: 1.57 mm; outer diameter: 2.41 mm) containing 17β-estradiol (estradiol) subcutaneously implanted between the shoulder blades and neck. The dose of estradiol (E8875, Sigma-Aldrich) was based on previous publications and estimated to give levels similar to estrus/diestrus for the Ovx–low estradiol (Ovx^LowE^) group and proestrus (or higher) for the Ovx–high estradiol (Ovx^HighE^) group (Desroziers et al., 2017; Hellier et al., 2018; Porteous et al., 2021). Ovx^LowE^ mice received an implant with 4 µg estradiol dissolved in absolute ethyl alcohol and mixed with silastic gel. Ovx^HighE^ mice received an implant containing crystalline estradiol mixed 1:1 with cholesterol. One group of mice were Ovx and received an implant containing only cholesterol. All mice were left for 2–3 weeks before being used for tissue collection or electrophysiology.

### Blood, tissue collection and ELISA

All mice were habituated to handling for at least 4 days prior to tissue collection. Mice were euthanized (between 09:00 and 11:00 h) and trunk blood was collected in tubes. All blood samples were kept on ice before being centrifuged. Uterus and adrenal glands were dissected out and weighed immediately following decapitation. Uterus masses were also taken from a subset of animals used for electrophysiology; the protocol for dissection and weighing remained the same. Adrenal gland mass is the combined mass of both left and right adrenals for each animal. Thymus glands were dissected out and stored in 4% PFA before being weighed. Serum corticosterone was measured using an ELISA (Arbor Assays, catalogue no. K014,RRID AB_2877626) according to the manufacturer's instructions.

### Slice preparation

Mice were killed by cervical dislocation between 09:00 and 11:00 h, their brain was quickly removed and placed in ice-cold oxygenated (95% O_2_, 5% CO_2_) slicing solution containing (in mmol l^−1^): 87 NaCl, 2.5 KCl, 25 NaHCO_3_, 1.25 NaH_2_PO_4_, 0.5 CaCl_2_, 6 MgCl_2_, 25 d-glucose and 75 sucrose, pH 7.2–7.4. A vibratome (VT1200S, Lecia Microsystems) was used to cut 200-µm-thick coronal slices of the PVN, which were then incubated in oxygenated artificial cerebrospinal fluid (aCSF) containing (in mmol l^−1^): 126 NaCl, 2.5 KCl, 26 NaHCO_3_, 1.25 NaH_2_PO_4_, 2.5 CaCl_2_, 1.5 MgCl_2_ and 10 d-glucose at 30°C for at least 1 h before recording. For recording, slices were transferred to a recording chamber and continuously perfused with 30°C aCSF at 1.5 ml min^−1^. CRH neurons within the PVN were visualized using a 40× objective and epifluorescence to excite tdTomato.

### Whole-cell electrophysiology recordings

Electrophysiological recordings were collected with a Multiclamp 700B amplifier (Molecular Devices), filtered at 2 kHz, and digitized using the Digidata 1440a (Molecular Devices). Data were analysed with Clampfit 10.7 (Molecular Devices).

For whole-cell recordings, borosilicate glass pipettes (tip resistance: 2–5 MΩ) were filled with an internal solution containing (in mmol l^−1^): 120 K-gluconate, 15 KCl, 0.5 Na_2_EGTA, 2 Mg_2_ATP, 0.4 Na_2_GTP, 10 HEPES, 5 Na_2_-phosphocreatine and 0.25% neurobiotin (adjusted to pH 7.2 with KOH; adjusted to ≈290 mOsm with sucrose). All current clamp experiments were performed in the presence of 10 μmol l^−1^ cyanquixaline (6-cyano-7-nitroquinoxaline-2,3-dione) (CNQX) and picrotoxin (50 μmol l^−1^). Each cell was held at approximately −60 mV. The liquid junction potential was calculated to be approximately −14.1 mV and was not compensated for. Cells were not recorded from if input resistance was below 0.7 GΩ or access resistance was above 30 MΩ and both input and access resistance were monitored throughout to ensure stable recording. We used a current step protocol to determine spike output and first spike latency (FSL). The step protocol consisted of 300 ms square steps from 0 to +50 pA in 5 pA increments. Spikes were detected using a threshold search in Clampfit and were analysed for rise time, decay time, amplitude and half width. FSL was calculated from the time of the depolarizing step initiation to the action potential (AP) threshold for the first spike evoked at steps equal or greater than 10 pA. AP threshold was defined as the voltage at which the AP first derivative crossed 10 mV ms^−1^. The same analysis criteria were used to identify FSL and AP threshold for a 1 s, +40 pA s^−1^ ramp protocol.

For all voltage clamp recordings, neurons were clamped at −60 mV. Input resistance, access resistance and capacitance were monitored periodically throughout recordings. *I*_A_ current recordings were performed in the presence of CNQX (10 μmol l^−1^), picrotoxin (50 μmol l^−1^), tetrodotoxin (TTX; 0.5 μmol l^−1^), XE991 (40 μmol l^−1^) and nifedipine (100 μmol l^−1^). To evoke *I*_A_ currents, neurons were hyperpolarized from −60 to −110 mV for 500 ms before a family of depolarizing steps were delivered in 10 mV steps from −100 to +30 mV. Peak *I*_A_ amplitude for each voltage step was measured and normalized to capacitance to give the current densities (pA pF^−1^).

A protocol was used to measure the *I*_M_ relaxation current, similar to that used in previous studies (Hu et al., 2016; Roepke et al., 2011). These recordings were performed in the presence of CNQX (10 μmol l^−1^), picrotoxin (50 μmol l^−1^) and TTX (0.5 μmol l^−1^). This protocol consisted of a pre-pulse to −20 mV for 300 ms followed by 500 ms steps from −30 to −75 mV. The *I*_M_ relaxation current was measured as the amplitude difference between the initial current and the sustained current at the end of the voltage step.

### Analysis

Statistical analysis was performed using GraphPad Prism 8. All reported values are means±s.e.m. Comparisons between groups were carried out using either one- or two-way ANOVA where appropriate, with Tukey’s *post hoc* multiple comparison tests. All *n*-values represent neuron number, all groups had *N*>3 animals. *P*<0.05 was considered statistically significant. *P*-values reported on figures are for *post hoc* multiple comparison tests.

## RESULTS

### Chronic estradiol effects on uterine mass, adrenal mass and corticosterone

**Fig. 1.**
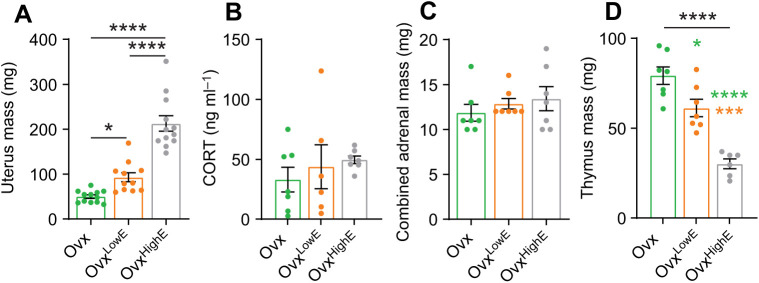
**Consequences of chronic estradiol treatment.** (A) Uterus mass for each group measured immediately after the animals were euthanized: Ovx (green), Ovx^LowE^ (orange) and Ovx^HighE^ (grey). Asterisks indicate significance identified by Tukey's multiple comparisons. Both low and high chronic estradiol implants increase uterus mass compared with Ovx animals. (B) There was no significant difference in circulating serum CORT levels taken from trunk blood samples. (C) Comparison of combined (mass of both left and right adrenal) adrenal mass between the groups. There was no significant difference in adrenal mass between the groups. (D) There was a significant difference in thymus mass between the three groups. Black asterisks indicate significant result of one-way ANOVA, coloured asterisks indicate *post hoc* Tukey's multiple comparisons test. Green asterisks indicate significance with the Ovx group, orange asterisks indicate significant difference with the Ovx^LowE^ group. *N*>6 mice for all groups. *P*-values: *P≤0.05, ***P≤0.001, ****P≤0.0001.

In order to manipulate estradiol levels, female mice were ovariectomized and either received no treatment (Ovx) or received a low (Ovx^LowE^) or high (Ovx^HighE^) dose estradiol implant. Two to three weeks later, animals were euthanized and blood and tissue were collected. The uterus is highly sensitive to estradiol, shows enlargement in response to estradiol elevations and has been previously used as a bioassay for estrogen levels (Owens and Ashby, 2002; Serova et al., 2010). Estradiol treatment induced a significant increase in uterine mass (one-way ANOVA, *F*_2,32_=51.91, *P<*0.0001; Fig. 1A), consistent with previous studies. Previous work has shown that estradiol treatment can also elevate basal corticosterone levels in rats (Figueiredo et al., 2007; Kitay, 1963; Lo et al., 2000), but have no effect (Aoki et al., 2010; Speert et al., 2002; Wada et al., 2018) or reduce corticosterone levels in mice (Daodee et al., 2022; Eid et al., 2020; Tantipongpiradet et al., 2019). Here, we found that in Ovx mice, high or low dose estradiol implants did not significantly change morning corticosterone levels (one-way ANOVA, *F*_2,17_=0.55, *P*=0.58; Fig. 1B). Likewise, combined adrenal mass was also not different across the estradiol treatment groups (one-way ANOVA, *F*_2,18_=0.64, *P*=0.54; Fig. 1C). Interestingly, thymus mass, which can be influenced by both corticosterone (Karatsoreos et al., 2010) and estradiol (Clarke and Kendall, 1989; Utsuyama and Hirokawa, 1989; Zoller and Kersh, 2006), was significantly different between the groups (one-way ANOVA, *F*_2,17_=30.52, *P*<0.0001; Fig. 1D). *Post hoc* Tukey's multiple comparisons showed a significant difference between Ovx^HighE^ and both Ovx and Ovx^LowE^ (*P*<0.0001 and *P*=0.0004, respectively), and a significant difference between Ovx and Ovx^LowE^ (*P*=0.023).

### Estradiol regulates *I*_A_ potassium channel currents in CRH neurons

**Fig. 2.**
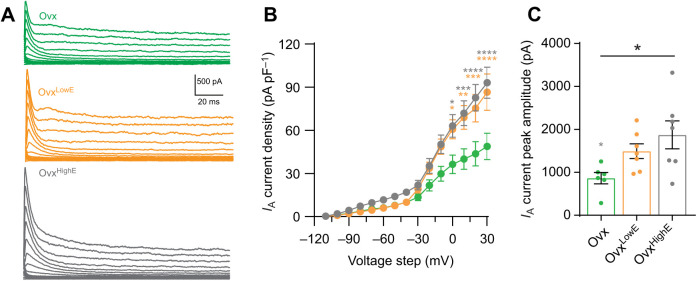
***I*_A_ currents are influenced by chronic estradiol treatment.** (A) Evoked *I*_A_ currents from individual corticotropin releasing hormone (CRH) cells in each group: Ovx (green), Ovx^LowE^ (orange) and Ovx^HighE^ (grey). (B) *I*_A_ current densities plotted for each 10 mV voltage step from −100 to +30 mV. Cells from Ovx animals had significantly smaller *I*_A_ current densities compared with cells from Ovx^LowE^ and Ovx^HighE^. (C) Peak amplitude *I*_A_ currents (not normalized to capacitance) evoked by a +30 mV step. Peak *I*_A_ currents in Ovx animals were significantly smaller than in Ovx^LowE^ and Ovx^HighE^. Results of one- and two-way ANOVAs are reported in Table 2. Asterisks denote significance by Tukey's multiple comparisons test: orange, Ovx^LowE^ versus Ovx; grey, Ovx^HighE^ versus Ovx. There were no significant differences between the Ovx^LowE^ and Ovx^HighE^ groups. *N*=3–5 mice for all groups. *P*-values: *P≤0.05, **P≤0.01, ***P≤0.001, ****P≤0.0001.

Neuronal intrinsic excitability is dictated in part by voltage-gated ion channel density and function. We have previously shown that *I*_A_, a transient K^+^ current, is regulated over the estrous cycle in CRH neurons (Power and Iremonger, 2021). To investigate the link between estradiol levels and *I*_A_ currents, we used a voltage clamp protocol on CRH neurons from Ovx, Ovx^LowE^ or Ovx^HighE^ manipulated mice. Electrophysiological recordings were performed 2–3 weeks post ovariectomy. A two-way repeated-measures (RM) ANOVA revealed that there was a significant effect of estradiol treatment on *I*_A_ current density (*F*_2,18_=5.32, *P*=0.015; Fig. 2A,B), a significant effect of voltage step (*F*_14,252_=134.1, *P*<0.0001) and a significant interaction (*F*_28,252_=4.68, *P*<0.0001). *Post hoc* tests showed that current densities at multiple voltage steps were smallest in Ovx animals compared with Ovx^LowE^ (*P*<0.05) and Ovx^HighE^ animals (*P*<0.05). Peak amplitude of the current at the maximum voltage step (+30 mV) was also significantly different between groups (one-way ANOVA, *F*_2,17_=4.55, *P*=0.026; Fig. 2C). *Post hoc* comparison revealed a significant difference between Ovx and Ovx^HighE^ (*P*=0.021) but not with Ovx^LowE^ (*P*=0.18). These findings show that chronic estradiol manipulations lead to changes in *I*_A_ K^+^ currents in CRH neurons.

### Estradiol regulates *I*_M_ potassium channel currents in CRH neurons

**Fig. 3.**
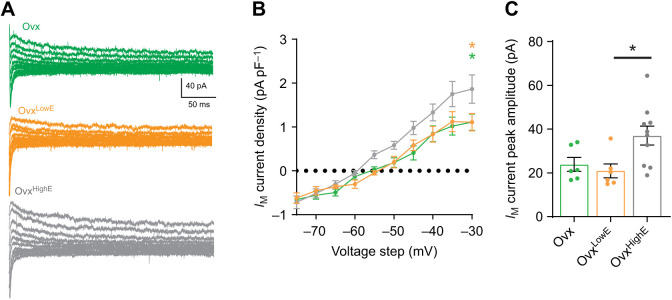
***I*_M_ currents are influenced by high chronic estradiol levels but not low.** (A) Evoked *I*_M_ currents from individual CRH cells in each group: Ovx (green), Ovx^LowE^ (orange) and Ovx^HighE^ (grey). (B) *I*_M_ current densities plotted for each 5 mV voltage step from −75 to −30 mV. Cells from Ovx^HighE^ animals had significantly larger *I*_M_ current densities compared with cells from Ovx^LowE^ and Ovx. (C) Peak amplitude *I*_M_ currents (not normalized to capacitance) evoked by a −30 mV step. Peak *I*_M_ currents in Ovx^HighE^ animals were significantly larger than in Ovx^LowE^, but not compared with cells from Ovx animals. Results of one- and two-way ANOVAs are reported in Table 2. Asterisks denote significance by Tukey's multiple comparisons test: orange, Ovx^LowE^ versus Ovx^HighE^; green, Ovx versus Ovx^HighE^. There were no significant differences between the Ovx^LowE^ and Ovx groups. *N*=3–5 mice for all groups. *P*-values: *P≤0.05.

In addition to *I*_A_, M-type (*I*_M_) potassium currents were also investigated. *I*_M_ currents are slowly activating, non-inactivating voltage-gated currents. They can contribute to intrinsic excitability via regulation of resting membrane potential and are ubiquitously found in neurons (Gutman et al., 2005). A voltage clamp protocol was used to measure the relaxation of the *I*_M_ current (see Materials and Methods). Comparison of the three groups using a two-way RM ANOVA revealed a significant effect of chronic estradiol treatment on *I*_M­_ current densities (*F*_2,19_=5.37, *P=*0.014; Fig. 3A,B). *Post hoc* multiple comparisons revealed that the Ovx^HighE^ group had a significantly higher *I*_M_ current density compared with both Ovx (*P*=0.04) and Ovx^LowE^ (*P*=0.04) at the highest voltage step (–30 mV). A one-way ANOVA comparing peak *I*_M­_ current amplitude in the three groups was also significant (*F*_2,19_=5.01, *P*=0.018; Fig. 3C), with multiple comparisons revealing significant differences between Ovx^HighE^ and Ovx^LowE^ (*P*=0.026) but not between Ovx^HighE^ and Ovx (*P*=0.075). These results show that estradiol levels also regulate *I*_M_ K^+^ channel currents in CRH neurons.

### Chronic estradiol manipulations do not alter CRH neuron intrinsic excitability

**Fig. 4.**
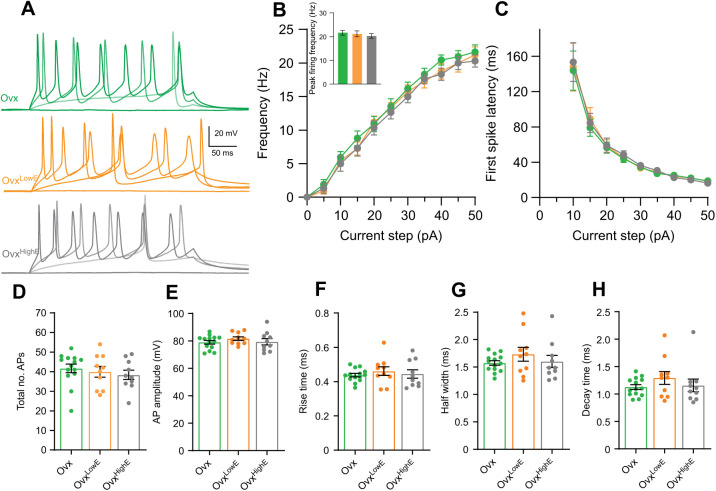
**Chronic estradiol does not change CRH neuron intrinsic excitability or action potential (AP) parameters.** (A) Representative responses of CRH neurons to 0, 10, 30 and 50 pA current steps of the frequncy–current (*F*–*I*) curve in each group: Ovx (green), Ovx^LowE^ (orange) and Ovx^HighE^ (grey). (B) Summary data for the *F*–*I* curve. There was no significant difference between the three groups across the *F*–*I* curve or at peak firing frequency (50 pA step, inset). Results of two-way ANOVA are reported in Table 2. (C) First spike latency (FSL) for each current step. There was no significant difference between the groups. Results of two-way ANOVA are reported in Table 2. (D) Total number of APs fired over all current steps for each group. There was no significant difference between the groups. All AP parameters were measured from the first AP fired from each cell. There was no significant difference between the groups in AP amplitude (E), rise time (F), half width (G) or decay time (H). Results of one-way ANOVA are reported in Table 2.

As both *I*_A_ and *I*_M_ currents are altered by artificially induced estradiol concentrations, we next investigated whether CRH neuron intrinsic excitability was also influenced. Given the elevated K^+^ currents in CRH neurons from Ovx^LowE^ and Ovx^HighE^ mice, we expected lower intrinsic excitability levels from these neurons compared with those from Ovx animals. Neurons were held around −60 mV in current clamp before injecting a family of current steps from 0 to +50 pA in 5 pA increments (Fig. 4A,B). This protocol was used to generate a frequency–current curve (*F*–*I* curve) and was performed on CRH neurons from Ovx, Ovx^LowE^ and Ovx^HighE^ animals. CRH neuron firing frequency was not different between the groups (two-way RM ANOVA, *F*_2,32_=0.386, *P*=0.61; Fig. 4A,B, Table 1), nor was peak firing frequency (one-way ANOVA, *F*_2,27_=3.102, *P*=0.68; Fig. 4B inset), or the slope of the *F*–*I* curves (one-way ANOVA, *F*_2,32_=0.19, *P*=0.83; Table 1). FSL, measured from the 10 pA current step onwards, was not affected by chronic estradiol treatment (two-way RM ANOVA, *F*_2,31_=0.059, *P=*0.94; Fig. 4C, Table 1). The total number of APs fired over all current steps was also similar between groups (one-way ANOVA, *F*_2,30_=0.17, *P*=0.84; Fig. 4D). Analysis of AP parameters showed no significant differences in amplitude, rise time, half width or decay time between the three groups (Fig. 4E–H, see Table 1 for mean values and Table 2 for statistics). There were also no significant differences in capacitance or input resistance between groups (one-way ANOVA, *F*_2,85_=1.93, *P=*0.15, and *F*_2,85_=1.6, *P=*0.21, respectively; Tables 1 and 2).

**Table 1.**
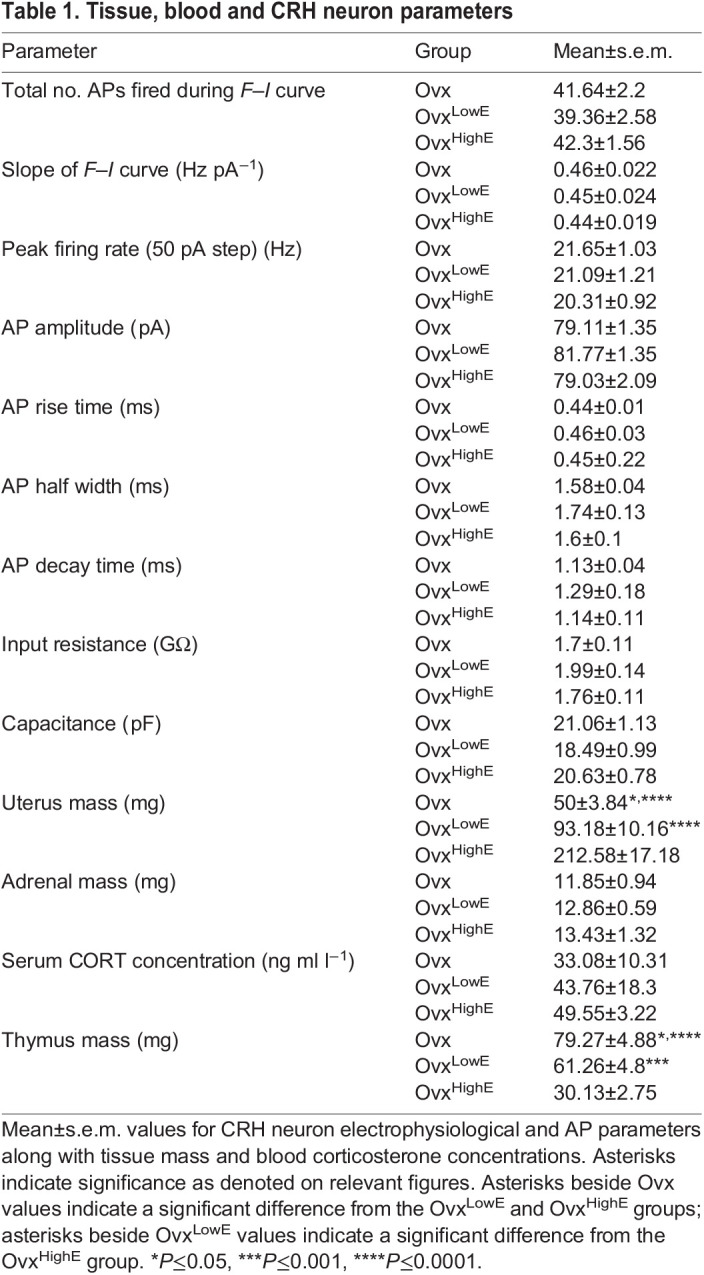
Tissue, blood and CRH neuron parameters

**Table 2.**
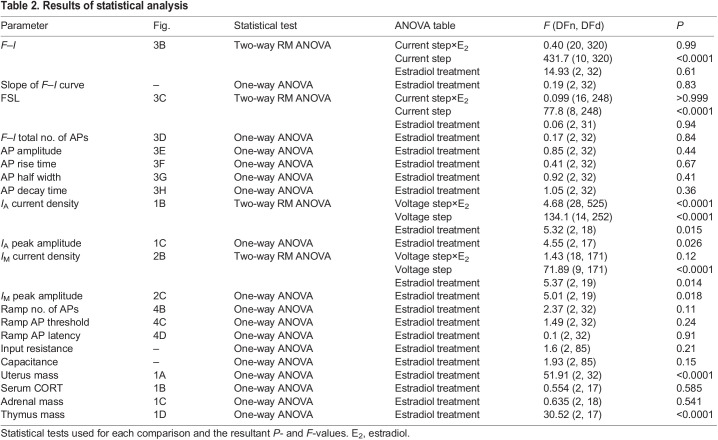
Results of statistical analysis

**Fig. 5.**
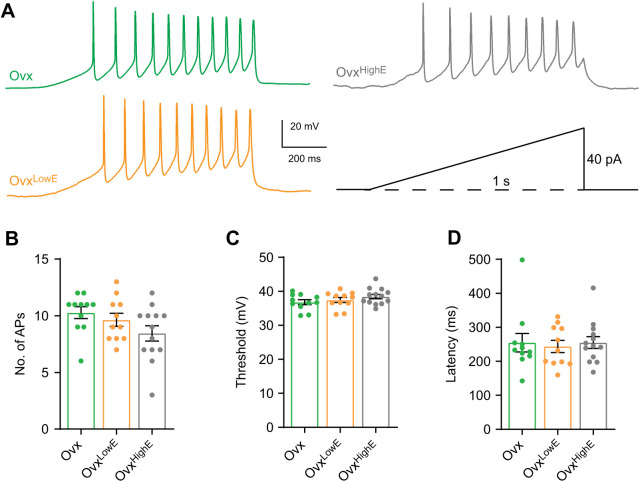
**Chronic estradiol does not influence CRH neuron intrinsic excitability measured by a current ramp.** (A) Example traces showing CRH neuron spiking response to a 1 s, 40 pA, current ramp protocol (bottom right) in each group: Ovx (green), Ovx^LowE^ (orange) and Ovx^HighE^ (grey). (B) There was no significant difference in the total number of APs fired during the ramp protocol between the three groups. (C) Neither high nor low estradiol concentrations altered AP threshold compared with Ovx. AP threshold was defined as the voltage at which the AP first derivative crossed 10 mV ms^−1^. (D) There was no change in AP latency between the groups. Results of one-way ANOVAs are reported in Table 2.

In addition to an *F*–*I* curve, CRH neuron excitability was also tested using a current clamp ramp protocol consisting of a 40 pA ramp delivered over 1 s (Fig. 5A). This protocol gives a more accurate measurement of latency to first spike and AP threshold compared with measurements from *F*–*I* curves. Neither the number of APs fired (Fig. 5B), AP threshold (Fig. 5C) or FSL (Fig. 5D) were significantly different between the groups (one-way ANOVA, *F*_2,29_=1.89, *P*=0.17, *F*_2,31_=0.64, *P*=0.54, and *F*_2,31_=0.22, *P*=0.80, respectively). Despite chronic estradiol treatment causing changes in K^+^ channel function, these results show that chronic estradiol manipulation had no impact on CRH neuron intrinsic excitability.

### *I*_A­_ currents correlate with CRH neuron excitability

**Fig. 6.**
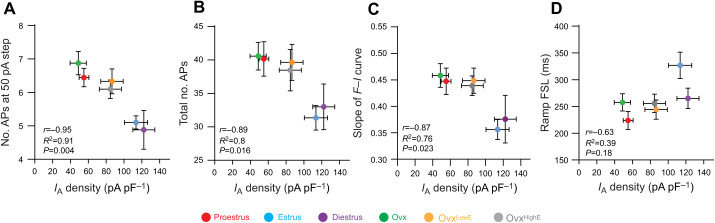
***I*_A_ current densities correlate strongly with measures of CRH neuron excitability.** (A) Correlation analysis between the number of APs fired at the highest (50 pA) current step given during the *F*–*I* curve and peak *I*_A_ current densities. Red is proestrus, blue is estrus, purple is diestrus, green is Ovx, orange is Ovx^LowE^ and grey is Ovx^HighE^. (B) *I*_A_ current densities versus total number of APs fired over the entire *F*–*I* curve for each group. (C) The slope of the *F*–*I* curve was correlated with *I*_A_ current densities. (D) FSL of current ramp protocol correlated with *I*_A_ current densities. *r*, *R^2^* and *P*-values for each comparison are listed on individual graphs.

We have previously shown that *I*_A_ currents are regulated over the female estrous cycle and control CRH neuron intrinsic excitability (Power and Iremonger, 2021). We took the data from this previous work along with data from the present study and performed Pearson's correlation tests for *I*_A_ current density versus various parameters of excitability measured from *F*–*I* curves and ramp protocol (Fig. 6). We included data from the following groups: intact estrus, intact proestrus, intact diestrus, Ovx, Ovx^LowE^ and Ovx^HighE^.

*I*_A_ current densities were found to be negatively correlated with the number of APs fired at the 50 pA current step (*r*=0.95, *P*=0.002; Fig. 6A), the total number of APs fired (*r*=0.89, *P*=0.016; Fig. 6B) and the slope of the *F*–*I* curve (*r*=0.87, *P*=0.023; Fig. 6C). *I*_A_ current densities were also positively correlated with current ramp FSL, although this was not significant (*r*=0.63, *P*=0.18; Fig. 6D). These data show that changes in *I*_A_ current density in CRH neurons are correlated with several parameters of intrinsic excitability.

## DISCUSSION

Circulating levels of estradiol have been previously shown to regulate the HPA axis (Patchev et al., 1995; Roy et al., 1999; Figueiredo et al., 2007); however, the impact of estradiol on CRH neuron excitability has been less clear. In the present study, we found that compared with Ovx mice, replacement with either low or high doses of estradiol increased *I*_A_ current density in CRH neurons. For *I*_M_ currents, only high estradiol concentrations led to an increase in current density. Despite these changes in K^+^ currents following estradiol manipulations, there were no significant changes in intrinsic excitability parameters. However, when we combined data from the present study with that of previous work looking at excitability in CRH neurons from intact, cycling females, we found significant correlations between *I*_A_ current density and several measures of excitability.

These findings differ compared with previous studies investigating estradiol effects on K^+^ currents in hypothalamic neurons. Hu et al. (2016) demonstrated that acute bath application of estradiol (100 nmol l^−1^, 10 min) onto CRH neurons from Ovx mice could supress *I*_M­_ currents. This effect could be replicated with a membrane-associated estrogen receptor (ER) agonist, suggesting that the fast suppression of M currents by estradiol was mediated via a non-genomic signalling mechanism (Hu et al., 2016). In other neural populations, chronic estradiol replacement in Ovx animals has also been shown to suppress K^+^ currents. In rostral ventrolateral medulla projecting preautonomic PVN neurons, estradiol treatment in Ovx rats was sufficient to reduce *I*_A_ current density (Lee et al., 2013). In GnRH neurons, estradiol treatment in Ovx mice also decreased both *I*_A_ and *I*_K_ currents (DeFazio and Moenter, 2002). Previously, we have shown that *I*­_A_ currents in CRH neurons are smallest during the proestrus phase and largest during the estrus stage of the mouse estrous cycle (Power and Iremonger, 2021). However, hormone profiles in intact animals will be different compared with those in Ovx animals with estradiol replacement, and this may underlie the differing findings.

What signalling pathways could be responsible for the effects of estradiol on K^+^ channel function in CRH neurons in the present study? Estradiol acts through two main receptors, ERα and ERβ. In Ovx rats, these receptors have opposing effects on stress induced glucocorticoid secretion, with ERα increasing secretion and ERβ decreasing it (Weiser and Handa, 2009; Weiser et al., 2010). ERβ is expressed in PVN neurons and shows colocalization with CRH (Lund et al., 2006; Oyola et al., 2017). Therefore, estradiol acting through ERβ could possibly be mediating the effects observed. Comparatively, ERα shows little to no expression in mouse PVN CRH neurons (Suzuki and Handa, 2005); however, it may regulate CRH neuron function indirectly via afferent inputs (Dayas et al., 2000). There are a number of different neural populations that express ERα and project to the PVN including neurons in the arcuate nucleus (Franceschini et al., 2006; Handa and Weiser, 2014), the bed nucleus of stria terminalis (Shughrue et al., 1997) and the peri-PVN region (Weiser and Handa, 2009). In addition, estradiol manipulations are known to regulate the signalling of other neurotransmitter systems in the PVN including serotonin (McAllister et al., 2012), oxytocin (Amico et al., 1981) and vasopressin (Lagunas et al., 2019; Vilhena-Franco et al., 2019). In summary, because estradiol modulates a number of neural circuits, neurotransmitter systems and hormone systems, it is likely that changes in CRH neuron function result from a combination of direct and indirect effects of estradiol. Although the relative importance of each of these pathways for mediating the changes in K^+^ channel function in CRH neurons is currently unclear, we can conclude that the initial trigger for these changes is the change in circulating estradiol.

Despite changes in K^+^ channel activity, chronic estradiol manipulations did not influence specific parameters of CRH neuron intrinsic excitability. However, the correlation analysis showed that there is a significant relationship between the *I*_A_ currents and CRH excitability when data from intact, cycling animals were included. Interestingly, although estradiol replacement increases *I*_A_ current density in Ovx^LowE^ and Ovx^HighE^ animals compared with Ovx, *I*_A_ current density does not reach the same level as that seen in intact diestrus or estrus mice. These data suggest that the magnitude of increase in K^+^ current density following estradiol replacement may not have been large enough to change CRH neuron intrinsic excitability.

A second reason why estradiol may not have impacted CRH neuron intrinsic excitability is homeostatic plasticity. Past research has shown that chronic manipulations of K^+^ channel function can induce compensatory changes in excitability known as homeostatic plasticity (Burrone et al., 2002). This form of plasticity acts to return the activity of neural circuits to a homeostatic set point (Burrone et al., 2002; Hengen et al., 2013, 2016; Keck et al., 2013; Turrigiano et al., 1998). CRH neurons may similarly have a ‘homeostatic setpoint’ firing rate, which in the intact animal is subject to a dynamically changing hormonal environment, resulting in temporary changes in excitability across the estrous cycle (Power and Iremonger, 2021). However, in a static hormonal environment, such as that seen in Ovx+estradiol treated mice, CRH neuron spiking excitability may retune to the setpoint despite differences in K^+^ channel activity. In order for this to happen, the function of other ion channels would need to be regulated. This hypothesis would be interesting to address in future work.

Together, data from the present study show that chronic estradiol elevations lead to enhanced K^+^ channel currents in CRH neurons. Although there were no obvious effects on spiking excitability, we predict that enhanced K^+^ channel function may affect how these neurons integrate and process stress-relevant synaptic inputs.
